# ST-segment elevation during transseptal puncture: A case report of a severe procedural complication

**DOI:** 10.1016/j.hrcr.2025.10.015

**Published:** 2025-10-17

**Authors:** Marisa van der Graaf, Isabelle N. Bax, Benno J.M.W. Rensing, Jippe C. Balt, Lucas V.A. Boersma

**Affiliations:** 1 Department of Cardiology, St. Antonius Hospital, Nieuwegein, The Netherlands; 2 Department of Cardiology, Amsterdam UMC, Amsterdam, The Netherlands

**Keywords:** Atrial fibrillation, Transseptal puncture, ST-segment deviations, Coronary artery spasms, Air embolism, Bezold-Jarisch reflex

**Key Teaching Points**

▪ Although complications during a transseptal puncture are uncommon, they can be life-threatening.
▪ ST-segment deviations can arise from different etiologies, including coronary artery spasm, air embolism, and the Bezold-Jarisch reflex.
▪ Differentiating between ST-segment deviation mechanisms is crucial, as treatment strategies differ.

## Introduction

Left atrial (LA) ablation is routinely performed in patients with atrial fibrillation (AF) who seek an alternative to antiarrhythmic drugs. Accessing the LA via transseptal puncture (TSP) is widely used and generally considered safe. However, this procedure is associated with a small but nonnegligible risk of complications (∼1%), most commonly cardiac tamponade.^1^, ^2^, ^3^ In some patients, transient ST-segment elevation may occur shortly after the TSP, which is usually considered to be due to a systemic air embolism, thromboembolic events, or a parasympathetic reflex.^2^

This report describes a case of a patient in whom ST-segment deviations occurred in relation to the TSP during 2 separate, consecutive ablation procedures. During the first procedure, the patient experienced profound cardiac arrest and hemodynamic shock, requiring 30 minutes of resuscitation before stabilization was achieved. This was initially assumed to be a rare event of coronary embolism, and a second procedure was planned. Despite additional precautions, generalized ST-segment deviations with hemodynamic deterioration occurred again, but resuscitation was averted through immediate intervention. This report discusses the mechanisms, the possible cause, and implications of this severe complication.

## Case report

A 61-year-old male patient, known with symptomatic persistent AF, was scheduled for an elective pulmonary vein isolation procedure. Except for a known history of hypertension, recovered tachycardiomyopathy, and left bundle branch block, the patient’s medical history was unremarkable. Preprocedural echocardiography showed a normal ejection fraction, mild mitral regurgitation, and a nondilated LA (LA volume index 28 mL/m^2^). Preprocedural magnetic resonance imaging was performed, showing no anatomical abnormalities.

**Figure 1:**
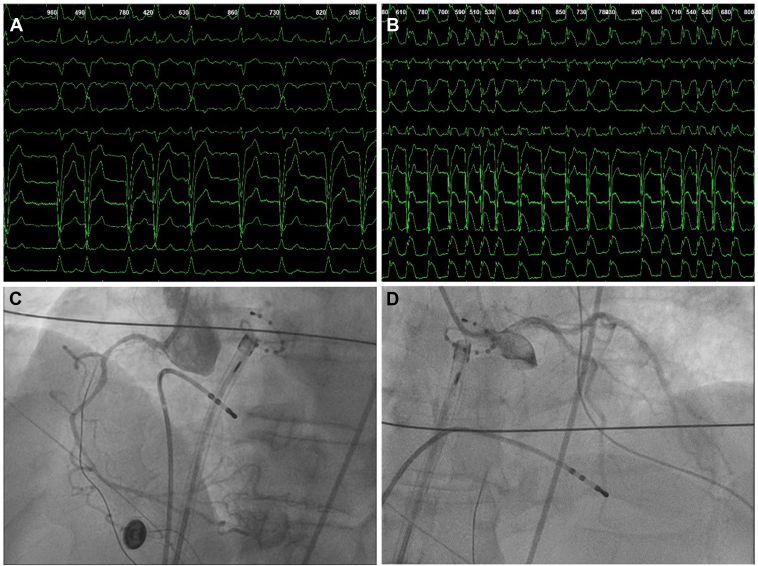
12-lead electrocardiogram (ECG) and coronary angiography recordings from the initial procedure. **A:** ECG recording demonstrating atrial fibrillation with left bundle branch block (LBBB) at the beginning of the procedure. **B:** ECG recording showing generalized ST-segment deviations a few minutes after the transseptal puncture. **C and D:** Urgent coronary angiography demonstrating no evidence of air embolism, coronary artery disease, or coronary spasms.

The ablation procedure was planned using pulsed field ablation technology and performed under general anesthesia with intravenous propofol, fentanyl, and rocuronium. After a successful TSP using a radiofrequency transseptal needle (NRG C1 RF, Boston Scientific Inc., MA), intravenous heparin and atropine were administrated. A circular mapping catheter was introduced into the LA through the FARADRIVE sheath to guide ablation. According to the instructions for use, the sheath was connected to a flush line and continuously perfused throughout the procedure. During LA mapping, a marked drop in blood pressure was observed, accompanied by widespread ST-segment deviations across all electrocardiogram (ECG) leads, shortly followed by cardiac arrest (Figure 1A and 1B). During resuscitation, alternating episodes of ventricular fibrillation and pulseless electrical activity were observed. Adrenaline and amiodarone were administered according to the resuscitation protocol. Transthoracic ultrasound was performed to exclude tamponade and pericardial effusion. Urgent coronary angiography (CAG) showed no signs of either coronary artery disease or air embolism (Figure 1C and 1D). After recovery of cardiac output and normalization of the ECG, the procedure was terminated and the patient was transferred to the intensive care unit. Postoperative transthoracic echocardiography showed a reduced left ventricular ejection fraction (45%–49%) with apicoseptal hypokinesis, while laboratory results demonstrated mildly elevated troponin and creatine kinase levels. The most likely cause of this incident was considered to be air embolism to the right coronary artery, presumed to have occurred during the introduction of the mapping catheter.^4^ The patient made a rapid recovery and was discharged from the hospital within a few days, without any signs of neurological damage.

Because of persistent AF despite moderately tolerated medical therapy, a second procedure was discussed with the patient and scheduled 4 months after the initial intervention. The need for resuscitation during the prior procedure was considered a rare but severe clinical complication, and a second procedure using pulsed field ablation under general anesthesia was subsequently planned. During the second procedure, the patient experienced recurrent hemodynamic instability, accompanied by diffuse ST-segment deviations across all leads immediately after the TSP using a Brockenbrough transseptal needle (BRK1, Abbott Vascular, CA, USA). After medical treatment, including high-dose inotropes and atropine, his blood pressure and ECG normalized, successfully avoiding cardiac arrest. An air embolism was considered extremely unlikely during this second procedure, since generalized ST-segment elevation occurred immediately after the TSP. The procedure was discontinued, and the patient was transferred to the cardiac care unit for postoperative monitoring. Postprocedural cardiac ultrasound was performed, which was comparable to preprocedural imaging. Three weeks after the second procedure, antiarrhythmic therapy was switched from sotalol to amiodarone, which was fairly tolerated. Although the frequency of AF episodes has markedly decreased, the patient remains symptomatic, and an optimal therapeutic solution has not yet been identified.

## Discussion

In this report, we present a case of recurrent severe hemodynamic instability and ST-segment deviations provoked by the TSP. During the first ablation procedure, this even progressed to cardiac arrest, with the initially suspected cause being coronary embolism. However, the course of events during the second procedure and reevaluation of the first procedure led us to hypothesize that a severe Bezold-Jarisch reflex was the most likely cause in both procedures. The different potential etiologies of ST-segment deviations and their clinical implications will be discussed below.

ST-segment elevation during the TSP can have various causes, such as the occurrence of an air embolism.^2^ While air embolisms are an uncommon complication of ablation procedures, they are presumably underrecognized because small embolisms may not cause ST-segment elevation.^5^ Adequate flushing of the sheath and catheters with saline before and during the ablation procedure is essential, and the use of continuous saline drip is recommended in international guidelines and consensus statements. If not performed properly, air may be introduced through the sheath and subsequently migrate into the circulation. Because the ostium of the right coronary artery is positioned relatively anteriorly and superiorly when a patient is lying supine, the air embolism tends to migrate into this artery, giving rise to ST-segment elevations in the inferior ECG leads.^4^^,^^6^

Another cause of ST-segment elevation during the TSP is the Bezold-Jarisch reflex. The atrial septum is innervated by parasympathetic nerves, which can be triggered or even damaged during the TSP and lead to the Bezold-Jarisch reflex. This parasympathetic reflex, activated by cardiac vagal afferents, inhibits sympathetic activity and causes symptoms such as bradycardia, hypotension, and vasodilation.^7^ The subsequent decrease in blood flow due to vasodilation may lead to ST-segment elevation. Since there are more cardiac vagal afferents in the right coronary artery, ST-segment elevation will be more pronounced in the inferior leads.^4^^,^^8^^,^^9^

Another cause of ST-segment elevation during the TSP, which should be taken into account, is the occurrence of coronary artery spasms (CAS). CAS during catheter ablation is rare but can lead to serious events, such as cardiogenic shock.^4^^,^^10^ CAS occurring before ablation (ie, during administering anesthetics, sheath insertion, and the TSP) accounts for approximately 21% of all CAS cases.^11^ Similar to the Bezold-Jarisch reflex, CAS after the TSP is influenced by the nervous system. During the TSP, cholinergic afferent fibers may be triggered, potentially because of a slightly malpositioned puncture site or as a result of fiber extensions within the fossa ovalis. The stimulation of these fibers enhances vagal activity, leading to the release of acetylcholine, which in turn may cause CAS.^4^^,^^8^ While CAS may be identified during urgent CAG, negative findings do not exclude CAS as the underlying cause of ST-segment elevation.^12^ An important difference between CAS and the Bezold-Jarisch reflex is that CAS leads to acute vasoconstriction of the coronary arteries and should therefore be treated with nitrates or calcium channel blockers.^13^ However, the Bezold-Jarisch reflex leads to vasodilation of the coronary arteries and should be treated with anticholinergic agents.^14^

A recent retrospective study reported a 0.31% incidence of transient ST-segment elevation in more than 2900 patients undergoing catheter ablation for AF.^10^ In most cases, these changes were observed directly after the TSP. While 6 patients had definite angiographic evidence of CAS, in 2 patients the ST-segment elevation resolved before urgent CAG could be performed and in 1 patient the ST-segment elevation normalized within 3 minutes, making urgent CAG unnecessary.^10^ These findings are comparable to another retrospective study, which reported a 0.38% incidence of transient ST-segment elevations.^4^ However, this may be an underestimation of the true incidence, as a prospective study by Vale et al^8^ reported ST-segment elevation in 5.2% of patients, with half of these cases being asymptomatic. These findings suggest that ST-segment elevations during the TSP can present with a wide spectrum of clinical severity, ranging from asymptomatic ECG changes to life-threatening events. While there is a substantial incidence of severe ST-segment elevations during the TSP requiring medical therapy and urgent CAG, previously published literature showed no cases in which this resulted in cardiac arrest.^3^^,^^4^^,^^8^^,^^15^

In our case, the ST-segment deviations during the first procedure were presumably detected at a relatively late stage (several minutes after the TSP) because of the presence of preexistent left bundle branch block. The longer time interval between the TSP and the onset of ST-segment elevation initially led to the suspicion of an air embolism. However, no air, thrombus, or other obstruction was observed during the CAG, which was performed 20 minutes after the initiation of resuscitation when the patient had become hemodynamically more stable.

During the second procedure, all personnel in the electrophysiology laboratory were aware of the prior events, so immediate measures were taken at the first signs of ST-segment elevation to prevent cardiac arrest. Nevertheless, because of persistent hemodynamic instability, proceeding with pulmonary vein isolation was deemed unsafe.

## Conclusion

In this case report, we describe a patient with recurrent hemodynamic deterioration after the TSP, most likely because of a severe Bezold-Jarisch reflex. In clinical practice, increased awareness is warranted regarding the potential occurrence of ST-segment elevations during or after TSP, including their different etiologies and clinical implications. Although ST-segment elevations are typically transient and asymptomatic, serious complications can occur if they are not promptly recognized and adequately treated.

## Disclosures

Dr Balt reports being consultant for Abbott. Dr Boersma reports being consultant/speaker/proctor for Boston Scientific and Medtronic and speaker for ZOLL, Biosense Webster, and Abbott. All fees go to the cardiology department. The rest of the authors have no conflicts of interest.
